# *Nox* Gene Expression and Cytochemical Localization of Hydrogen Peroxide in *Polyporus umbellatus* Sclerotial Formation

**DOI:** 10.3390/ijms141122967

**Published:** 2013-11-20

**Authors:** Yong-Mei Xing, Juan Chen, Chao Song, Ying-Ying Liu, Shun-Xing Guo, Chun-Lan Wang

**Affiliations:** Institute of Medicinal Plant Development, Chinese Academy of Medical Sciences & Peking Union Medical College, Malianwa North Road 151, Haidian District, Beijing 100193, China; E-Mails: meimary@163.com (Y.-M.X.); kibchenjuan@126.com (J.C.); songchao0423@163.com (C.S.); yyliuabc@gmail.com (Y.-Y.L.)

**Keywords:** *Polyporus umbellatus*, temperature shift, H_2_O_2_, *Nox*, SOD, CAT

## Abstract

The effect of temperature shift on *Polyporus umbellatus* sclerotial development was investigated. Micromorphology of the sclerotia was observed by using scanning electron microscopy (SEM). The cytochemical localization of H_2_O_2_ expressed as CeCl_3_ deposition at the subcellular level was observed by using transmission electron microscopy (TEM). *Nox* gene expression in sclerotia and mycelia was detected by quantitative real-time PCR (qRT-PCR) analysis. In addition, superoxide dismutase (SOD) and catalase (CAT) specific activities increased during sclerotial development and decreased after the antioxidant diphenyleneiodonium (DPI) was used. Results indicated that the temperature shift treatment induced *P. umbellatus* sclerotial formation. Compared with the mycelia, the *Nox* gene was respectively upregulated by 10.577-, 30.984- and 25.469-fold in the sclerotia of SI, SD and SM stages respectively. During the sclerotial formation, H_2_O_2_ accumulation was observed in the cell walls or around the organelle membranes of the mycelial cells. The antioxidant DPI decreased the generation of H_2_O_2_ in mycelial cells. The specific activity of SOD and CAT levels was decreased significantly by DPI. The activity of the two antioxidant enzymes in the mycelia increased much more during sclerotial formation (*p* < 0.05). Oxidative stress was closely associated with sclerotial development in *P. umbellatus* induced by temperature shift treatment.

## 1. Introduction

*Polyporus umbellatus* (Pers.) Fr. is one of the traditional medicinal mushrooms, of whose sclerotia exhibiting diverse pharmacological effects [1]. Due to a decreased abundance in natural sources, researchers have made great efforts to produce sclerotia in the laboratory.

Sclerotia are compact bodies of aggregated hyphae which can survive for long periods under unfavorable conditions such as starvation, coldness, nutrition depletion, *etc.* [2]. Environmental factors can affect fungal growth and sclerotial formation. It has been reported that the carbon source and growth medium initial pH affect the sclerotial formation of *P. umbellatus* [3]. Previously, we found that exposure to low temperatures increased the levels of reactive oxygen species (ROS) and rendered *P. umbellatus* sclerotial formation in a sawdust-based medium, so we preliminarily concluded that the sclerotial development was associated with oxidative stress [4–6].

NADPH oxidases (Nox) can generate ROS in eukaryotes. Nox is an important source of superoxide, including hydrogen peroxide [7]. It was well documented that both BC Nox A and B were involved in the sclerotial formation of *Botrytic cinerea* [8,9]. Therefore, NADPH oxidases residing in filamentous fungi have been drawn to the attention of researchers.

Filamentous fungi have to face oxidative stress throughout their lives, thus, the enzymatic and non-enzymatic antioxidant defense systems can protect organisms from the deleterious effects of ROS. SOD, which constitutes the first line of defense against ROS within a cell, is one of the most important enzymes to scavenge free radicals during oxidative stress and to encounter ROS in various biological systems; its function is to eliminate O_2_^−^[10]. On the other hand, by catalyzing H_2_O_2_ to generate H_2_O and O_2_[11], catalase (CAT) plays an important role in cellular antioxidant responses.

ROS might be closely related to *P. umbellatus* sclerotial formation induced by low temperature as shown previously [12], yet detailed information on oxidative stress and sclerotial morphogenesis at molecular levels remains unclear. Furthermore, the subcellular localization of ROS production has not yet been identified. Up to now, no transcriptomic or genomic data of *P. umbellatus* has been available. It has become urgent to conduct researches on the differentially expressed genes in *P. umbellatus* sclerotia and mycelia. Thus, *Nox* gene from *P. umbellatus* was cloned by using 3′ rapid amplification of cDNA end PCR (RACE). Although the sclerotia produced in the sawdust-based medium might be of more practical significance than those generated in the nutrient agar medium, it is difficult to explore the biological mechanisms of *P. umbellatus* sclerotial morphogenesis in such a medium of complicated composition. In this study, *P. umbellatus* sclerotia in nutritional agar medium were induced and screened for optimal conditions by temperature shift (low temperature) treatment.

The objective of this work is to further investigate the relationship between ROS and *P. umbellatus* sclerotial formation. Therefore, comparative observation on the micromorphology of the sclerotia induced by temperature shift treatment (from 25 °C to low temperatures) and the mycelia cultivated in the control group (cultured at 25 °C throughout the time) was performed by using SEM. In order to know more about ROS localization at the subcellular level, a cytochemical technique with CeCl_3_ reacting with H_2_O_2_ to generate the visible electron-dense deposits was used with TEM. As an effective means for reliable and rapid quantification of transcript levels with high specificity, sensitivity and broad dynamic range, qRT-PCR is widely used in the quantification of gene expression and in molecular diagnostics [13]. Therefore, transcriptional levels of the different expression of the *Nox* gene were examined by using qRT-PCR analysis in mycelia and sclerotia of *P. umbellatus* during the development stages. Moreover, the research was designed to evaluate the involvement of SOD and CAT in the response of mycelial cells to ROS during the process of *P. umbellatus* sclerotial formation.

The results obtained from this study may provide new insights into the mechanisms of *P. umbellatus* sclerotial formation.

## 2. Results and Discussion

### 2.1. Results

#### 2.1.1. *Polyporus umbellatus* Sclerotial Formation by Temperature Shift Treatment

**Figure 1:**
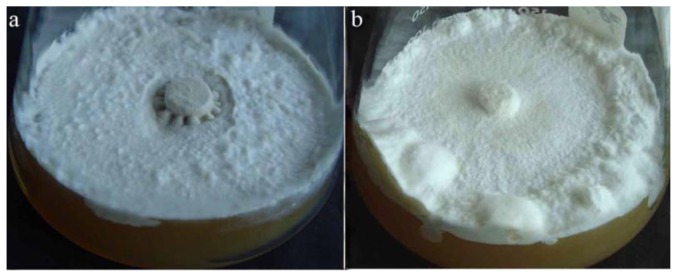

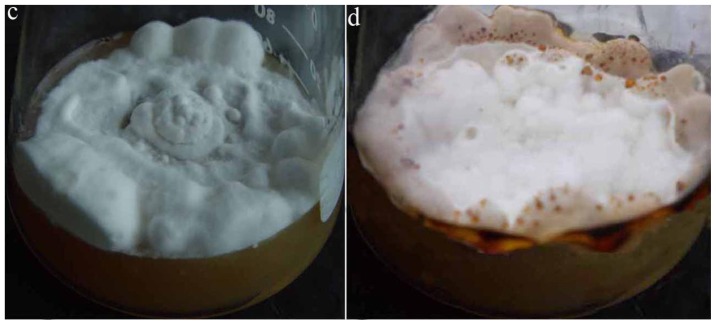
Different stages of sclerotial formation induced by low-temperature *vs.* mycelia in *Polyporus umbellatus* cultivated in the control group. (**a**) depicts *P. umbellatus* mycelia cultured at 25 °C without sclerotial development after cultivation for 120 days; (**b**) depicts SI stage of the *P. umbellatus* sclerotial formation cultivated at 8 °C with total cultivation for 60 days; (**c**) depicts SD stage at 8 °C after cultivation for 90 days; (**d**) depicts SM stage at 8 °C after cultivation for 120 days. Representative images were obtained from three independent experiments.

After being cultivated for about 30 days at 8 °C, *P. umbellatus* mycelia began to inflate into spherical, oval or irregular shapes, accompanied by interwoven hyphae in certain spots of the mycelium, which indicated the appearance of sclerotial initiation (SI) (Figure 1b). After being cultivated at low temperatures for 30 more days, the size of sclerotia initiation increased and the hyphae became further interwoven, which indicated the emergence of the sclerotial development (SD) stage (Figure 1c). After cultivation for 120 days in total, the sclerotia became even more condensed and most of the sclerotia turned black-brown, accompanied by black-brown droplets of pigment, which indicated that the sclerotial maturation (SM) stage had occurred (Figure 1d). However, the mycelia cultured at 25 °C (control group) throughout the time did not stimulate sclerotial development (Figure 1a).

#### 2.1.2. The Effect of NADPH Oxidase Inhibitor on Sclerotial Formation by Temperature Shift Treatment

**Figure 2:**
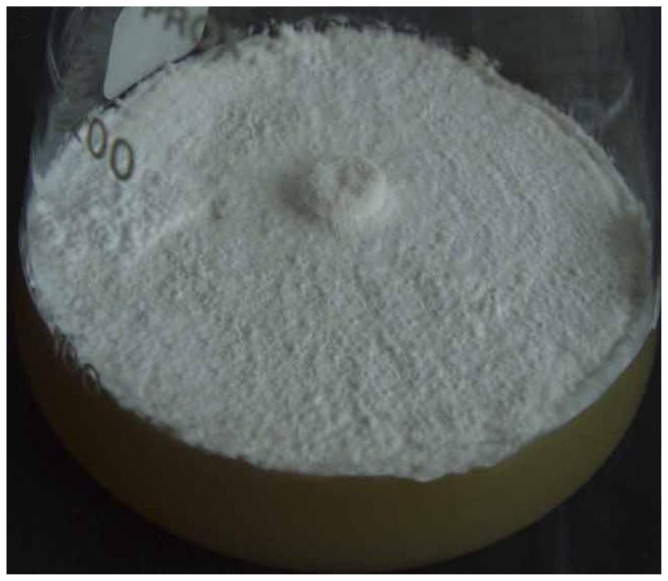
The effect of diphenyleneiodonium (DPI) (1 mmol L^−1^) on *P. umbellatus* sclerotial formation by the temperature shift treatment. No sclerotia were formed after 120 days of cultivation at 8 °C. Representative images were obtained from three independent experiments.

After the antioxidant diphenyleneiodonium (DPI) with different concentrations of 1 mmol L^−1^, 2 mmol L^−1^, 3 mmol L^−1^ respectively was added, no sclerotia were formed in each temperature shift group after cultivation for 120 days (Figure 2).

#### 2.1.3. SEM Examination of Artificial Sclerotia in the Temperature Shift Group and Mycelia in the Control Group after 120 days of Cultivation

**Figure 3:**
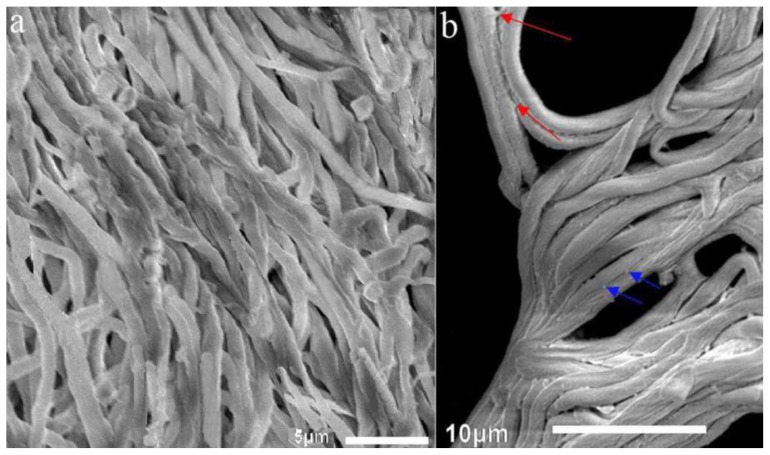
Scanning electron microscopy (SEM) examination of artificial sclerotia at 8 °C and mycelia at 25 °C after 120 days of cultivation. (**a**) depicts loosened mycelia cultured at 25 °C without sclerotial development; (**b**) depicts the condensed and fused mycelia in the SM stage of sclerotial formation cultivated at 8 °C. Images are representatives of three independent experiments. Scale bar, (**a**) 5 μm; (**b**) 10 μm.

Loosened mycelia in the control group which did not form sclerotia existed relatively independently. Hyphae fusion was seldom observed (Figure 3a). Mycelia in the temperature shift group in the SM stage of sclerotial formation became much more condensed than those in the control group (Figure 3b). We also found that hyphal adhesion could be observed during sclerotial formation in *P. umbellatus* (Figure 3b). In accordance with that stated by Erental in 2008 [14], the mucilage-like substance of filamentous shape (Figure 3b upper red arrows) or dot-like in size (Figure 3b lower blue arrows) turned up between the adjacent mycelia of *P. umbellatus* in the SM stage of sclerotial development. These substances seemed to be associated with hyphal adhesion and fusion. In addition, hyphal fusion and branching are probably the main processes during sclerotial formation. It was reported that hyphal strands tended to adhere together after coming into contact [15]. The same phenomenon occurred in the *P. umbellatus* sclerotial development in our current study.

#### 2.1.4. Cytochemical Localization of H_2_O_2_ Production Induced by Temperature Shift Treatment

**Figure 4:**
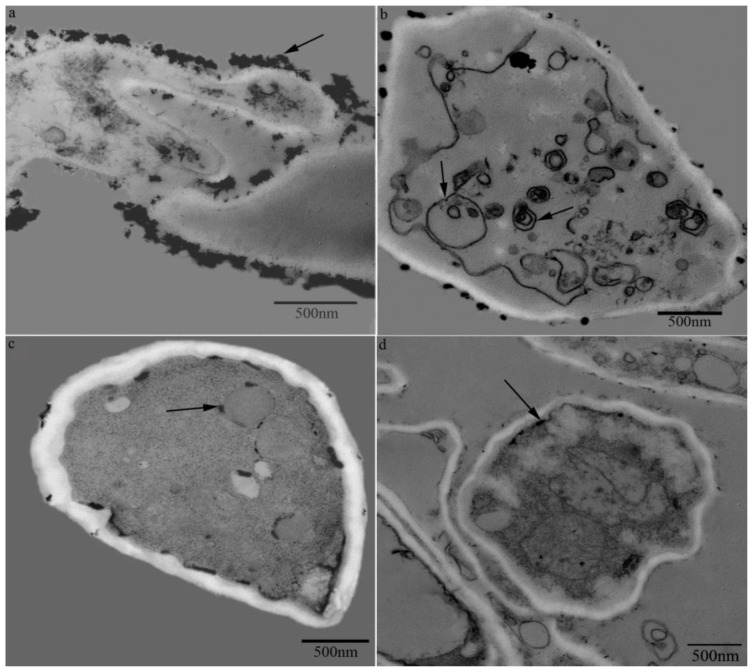
Cytochemical localization of H_2_O_2_ using transmission electron microscopy (TEM). (**a**) CeCl_3_ (*i.e.*, H_2_O_2_ production) is deposited around the cell walls (black arrow) in the SD stage of sclerotial formation; (**b**) H_2_O_2_ production is shown in the cell walls and around the organelles such as the tonoplasts and endoplasmic reticulum membranes (black arrows) during the SM stage of sclerotial formation; (**c**) Low concentrations of H_2_O_2_ are observed in the cell walls, tonoplasts and plasma membranes in the control group without sclerotial formation; (**d**) Low concentrations of H_2_O_2_ are observed in the cell walls and plasma membranes in the DPI treatment group. Pictures are representative of three independent experiments. Scale bar, 500nm.

In the control group, only a little CeCl_3_ was deposited in the cell walls, plasma membrane or tonoplasts of the mycelial cells, which indicated that only a small amount of H_2_O_2_ was produced in the control group (Figure 4c black arrow). In the temperature shift group of the SD stage during the sclerotial development, a large amount of H_2_O_2_ accumulation was observed mostly in the cell walls (Figure 4a). In the SM stage of sclerotial formation, much CeCl_3_ was deposited around the organelle membranes such as the tonoplasts and endoplasmic reticulum membranes, with some in the cell walls (Figure 4b black arrows). In the antioxidant group with DPI of 1 mmol L^−1^, little CeCl_3_ was observed in the cell walls, in the cytosol or in the internal organelle membranes of the mycelial cells (Figure 4d).

#### 2.1.5. *Nox* Gene Expression in Sclerotial Formation

**Figure 5:**
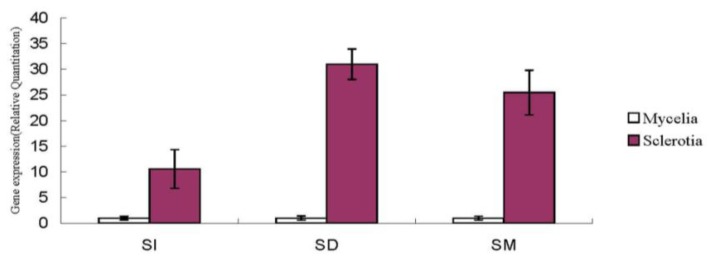
*Nox* gene expression during *P. umbellatus* sclerotial formation. The relative expression of *Nox* gene in the mycelia and the sclerotia was detected during the different stages of sclerotial formation (SI, SD and SM). Each symbol and vertical bar represent the mean values ± SD (*n* = 3).

A 2010-bp full-length cDNA of NADPH oxidase gene named *PUNOX* (Genebank accession number: Jk035912) contained 1674-bp ORF, and it was predicted to encode a 557 amino acid protein of 63,845 kDa, with an isoeletronic point (*PI*) of 5.58 [16]. In the SI stage of sclerotial formation, the *Nox* gene expression in sclerotia was 10.577 times that of the mycelia. In the SD stage, the *Nox* gene expression reached the maximum, which was 30.984 times that of the mycelia. In terms of the SM stage, the *Nox* gene was expressed 25.469 times that of the mycelia (Figure 5).

#### 2.1.6. Specific Activity of SOD during *P. umbellatus* Sclerotial Formation

**Figure 6:**
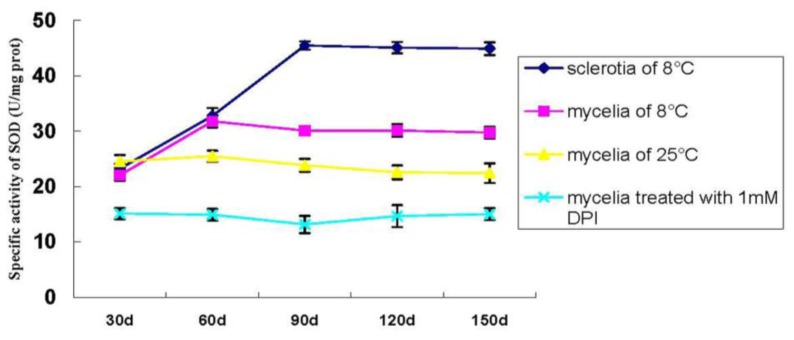
Specific activity of SOD during *P. umbellatus* sclerotial formation. Mean values ± SD or representatives of at least three independent experiments are shown (*n* = 10).

The specific activity of SOD in *P. umbellatus* mycelia cultivated at 25 °C throughout the time was stable and relatively low. The SOD level in mycelia was not high when cultured at 25 °C, but it was higher when exposed at 8 °C for a time of 60 days (SI) and retained this high level after that. The activity of SOD increased at a cultivation time of 90 days (SD) and remained stable at 120 days (SM) (Figure 6). After being treated with DPI (1 mmol L^−1^), sclerotia did not appear even in the temperature shift treatment and the SOD level was even lower than that of the mycelia cultivated at 25 °C throughout the cultivation (Figure 6).

#### 2.1.7. Catalase Activity during *P. umbellatus* Sclerotial Formation

**Figure 7:**
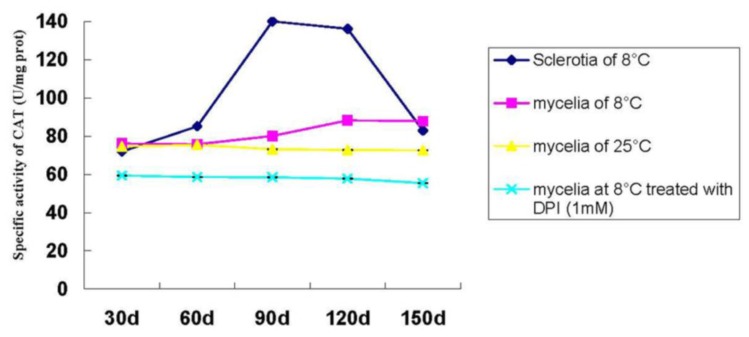
CAT activity during *P. umbellatus* sclerotial formation. Mean values ± SD or representatives of at least three independent experiments are shown (*n* = 10).

The CAT activity of the mycelia in *P. umbellatus* was low after 30 days of cultivation at 25 °C and the CAT activity of the sclerotia rose to a high level when the fungus was exposed to temperature shift treatment. Afterwards, it reached its highest level during the SD stage. However, it gradually decreased at the SM stage and dropped sharply after that (150 days). Moreover, it was found that the CAT activity was even lower than that of the mycelia cultivated at the same low temperature conditions. The CAT activity of the mycelia in *P. umbellatus* remained constant at 25 °C throughout the time (Figure 7). After being treated with the antioxidant DPI (1 mmol L^−1^), the CAT activity performed in a similar manner to the SOD activity during the *P. umbellatus* sclerotial formation (Figure 7).

### 2.2. Discussion

ROS is produced during the process of metabolism and plays an essential role in modulating fungal development [17]. A hyperoxidant condition can drive the fungal metabolic responses towards differentiation processes such as sclerotia, sexual structure formation, *etc.* [17]. It has been reported that the undifferentiated (growth) state is stable while the intermediate unstable state is the hyperoxidant state [6]. In the current research, ROS levels are relatively low under normal growth conditions (cultivated at 25 °C throughout the time), and generation and scavenging are kept balanced [18], which maintains intracellular O_2_ concentration below the level at which the generation of a hyperoxidant state is triggered [6]. However, during the stress (cultivated at 8 °C), the rate of ROS including H_2_O_2_ is dramatically elevated. It was documented previously that H_2_O_2_ is generated by *Sclerotium rolfsii* during sclerotial differentiation in response to the oxidizing growth factors of light and iron [19]. In the present study, we found that the low temperature treatment resulted in H_2_O_2_ accumulation during *P. umbellatus* sclerotial development and eventually induced its formation in the nutritional agar medium. The optimal temperature was 8 °C, but 5 °C was found better in sawdust-based medium [12]. The difference in the results might be attributed to the different compositions of the medium. Transferring from 25 °C to low temperature treatment provided *P. umbellatus* with hyperoxidative conditions. On the one hand, the solubility of O_2_ in water increases as the temperature decreases [19]. On the other hand, in case of the high oxidative stress state, the various antioxidant enzymes and other small molecules may play important roles in protecting *P. umbellatus* from the vicious effects derived from ROS [20]. As our study showed, compared with the mycelia cultivated in the control group, the SOD and the CAT specific activities of the mycelia cultured in the temperature shift group increased during the period of sclerotial initiation stage and reduced a little afterwards, and then maintained a high level throughout the sclerotial development process (Figures 5 and 6). This might be attributed to the fact that the equilibrium of oxidation and reduction reactions was destroyed and the ROS content correspondingly increased sharply. Thus, SOD and CAT rose to actively eliminate ROS generated in the times of stress.

During the *P. umbellatus* sclerotial formation, induced by temperature shift treatment, H_2_O_2_ accumulation deposited in the different sites of the mycelial cells. However, with the pretreatment with DPI, H_2_O_2_ production was counteracted partly with CeCl_3_ staining. Thus, the electron-dense deposits were seldom observed in the mycelial cells. The results of ROS generation at the subcellular level indicated that the *P. umbellatus* sclerotial development was accompanied by the production of ROS to some extent.

CAT was the endogenous factor for ROS scavenging, and it could eliminate intracellular H_2_O_2_[21]. The more free radicals that were produced, the higher the concentration of the antioxidant enzymes needed for their scavenging. After DPI was added to the medium, sclerotia could not be formed, even when treated at a low temperature. Meanwhile, the SOD and CAT activity of the mycelia in *P. umbellatus* decreased compared to that of the mycelia in the low temperature group. These results indirectly proved that sclerotial formation was closely connected to oxidative stress.

The qRT-PCR analysis of *Nox* gene in sclerotial formation revealed that *Nox* gene in the sclerotia was upregulated much more than that of the mycelia. Therefore, this was also probably involved in sclerotial formation of *P. umbellatus*. The information from this work may provide some important clues on the mechanisms of *P. umbellatus* sclerotial development under artificial conditions.

## 3. Experimental Section

### 3.1. Fungal Strain

The *P. umbellatus* used in this study was isolated from the wild triennial sclerotia collected from the Dangjiashan village, Beiping town, Gu County, Shanxi province, China by Professor Shun-Xing Guo. The cultures were maintained on wheat bran agar slants medium (wheat bran (30.0 g L^−1^), glucose (20.0 g L^−1^), KH_2_PO_4_ (1.0 g L^−1^), MgSO_4_ (1.0 g L^−1^), agar (10.0 g L^−1^)) in the dark at 4 °C. *P. umbellatus* had been identified previously by means of molecular biological identification of the internal transcribed region (ITS) of the 5.8S rDNA. Subsequently, *P. umbellatus* cultures were transformed from the wheat bran agar slants to a wheat bran substrate.

### 3.2. Reagent

Glucose, fructose, KH_2_PO_4_, MgSO_4_·7H_2_O, agar were purchased from Kebio Biotechnology Co., Ltd. in Beijing, China. CeCl_3_ and DPI were the products of Sigma Corporation (Santa Clara, CA, USA).

### 3.3. *P. umbellatus* Cultivation

The fructose complete medium was composed of fructose 20.0 g L^−1^, peptone 4.0 g L^−1^, MgSO^4^·7H^2^O 0.5 g L^−1^, KH^2^PO^4^ 0.5 g L^−1^, K^2^HPO^4^ 1.0 g L^−1^, vitamin B1 0.05 mg L^−1^ and agar 10.0 g L^−1^. The initial pH value was adjusted to 7.0 before the agar was added, and then the medium except for vitamin B1 was autoclaved. Vitamin B1 was sterilized by filtration through a 0.22 μm Millipore filter and then added evenly to the autoclaved medium [3]. The medium was poured into the conical flask. Mycelial plugs of 8 mm in diameter obtained from the actively-growing colonies in the petri dishes were transferred to the center of each conical flask containing 20 mL of solid agar medium at 25 °C under dark conditions.

### 3.4. The Effect of Temperature Shift on Sclerotial Formation in *P. umbellatus*

After *P. umbellatus* was cultivated at 25 °C for 30 days, the samples were then randomly divided into temperature shift group and control group. In the temperature shift group, we screened at the optimal conditions for *P. umbellatus* sclerotial formation. The fungus was respectively cultivated from 0 °C to 25 °C at one-degree increments, with 30 replicates in each group. The fungus growing at 25 °C throughout the time was considered to be the control group with 30 replicates. After screening, *P. umbellatus* cultured at 8 °C was found optimal for sclerotial formation. Therefore, we selected this condition for subsequent experiments, and the different stages of sclerotial development in *P. umbellatus* over time during cultivation were observed. Additionally, the experiment was repeated three times.

### 3.5. SEM Examination of the Sclerotia in the Temperature Shift Group and the Mycelia in the Control Group after Cultivation for 120 Days

The samples were prepared according to the protocol described previously [12]. The sclerotia in the temperature shift group and the mycelia in the control group were cultivated for 120 days and fixed with 2.5% glutaraldehyde for 48 h at 4 °C. Subsequently, the samples were air-dried and sputtered-coated with gold palladium, and then observed and photographed using a JEOL JSM-6510 Scanning Electron Microscope (Tokyo, Japan).

### 3.6. Cytochemical Localization of H_2_O_2_

A cytochemical technique with CeCl_3_ reacting with H_2_O_2_ to generate visible electron-dense deposits was used to investigate the subcellular localization of H_2_O_2_ accumulation induced by temperature shift treatment. Hydrogen peroxide of the mycelia in the sclerotia induced by temperature shift, the mycelia cultured throughout the time at 25 °C in the control group, and the mycelia cultivated with the antioxidant DPI (1 mM) by temperature shift treatment, were all visualized respectively at subcellular level using CeCl_3_ for cytochemical localization. Electron-dense CeCl_3_ deposited at the different sites of the cell with the presence of H_2_O_2_ detected by means of transmission electron microscope [22]. Tissue pieces of 2 mm_2_ in three different groups were incubated in freshly prepared 5 mM CeCl_3_ in 50 mM 3-(*N*-morpholino) propanesulfonic acid (Mops) with a pH value of 7.2 for about 1 h. The mycelia samples were then fixed in 2.5% glutaraldehyde in 0.1 M phosphate buffer, also with a pH value of 6.8 for 6 times, with the interval time for 30 min and postfixed in 1% osmium tetroxide for 24 h at 4 °C. After fixation, the samples were dehydrated in a range of ethanol from 30%–100% for 30min and then imbedded in the LR white resin. After that, the samples were polymerized for 48 h and cut using a diamond knife, and the ultrathin sections were collected on nickel nets. After being stained with uranyl acetate and citric acid, the sample sections were visualized by using JEM-1230 transmission electron microscope (Tokyo, Japan).

### 3.7. qRT-PCR Analysis of *Nox* Gene in Sclerotial Formation

The samples of the sclerotia in the SI, SD and SM stages and the mycelia cultivated in the same conical flask without sclerotial formation were respectively collected and immediately stored in liquid nitrogen. The mycelial samples were considered to be the control group, and the sclerotial specimens were regarded as the experiment group. The total RNA was extracted by using modified CTAB [23]. The full length of Nox cDNA was then cloned using 3′ rapid amplification of cDNA end PCR (RACE). Subsequently, reverse transcription reaction was performed according to MMLV Reverse Transcriptase (Promega, Madison, WI, USA) by using 2 μg of the total RNA of each sample. Consequently, the different expressions of *Nox* genes were detected by using qRT-PCR analysis. The 18S rRNA of *P. umbellatus* was selected for the reference gene. The cDNAs were diluted 1:40 in ddH_2_O. The gene-specific primers were designed by using primer 5. For 18S rRNA, 18S-FP: 5′-CCTTGTGCTGGCGATGCT-3′; 18S-RP: 5′-GCTGCCTTCCTTGGATGTG-3′. For NADPH oxidase (Nox), Nox-FP: 5′-TTTTCCTCCCACCCTCCTTG-3′; Nox-RP: 5′-CATTCAGCCTCTT ATTGGTTTCCT-3′. qRT-PCR was conducted using the SYBR green kit (TaKaRa, Dalian, China) and the ABI 7500 real-time PCR System (Applied Biosystems, Foster City, CA, USA). The reactions were started at 25 μL with 2 μL of cDNA template, 12.5 μL of SYBR^®^*Premix Ex Taq*™ Master Mix, 0.5 μL of each primer (10 μM), 0.5 μL of ROX Reference Dye and 9 μL of double distilled water. The thermal cycling conditions were as follows: 95 °C for 30 s; followed by 40 cycles of 95 °C for 15 s and 60 °C for 40 s. The relative expression was calculated as the ratio of the target gene expression normalized to 18S rRNA expression. All reactions, including the non-template controls, were carried out in triplicate. The cycle threshold (CT) values were generated by using ABI PRISM 7500 SDS Software v 1.4 (Applied Biosystems, Foster City, CA, USA) and the relative expression ratios were analyzed using the comparative DDC_T_ method of relative gene quantification [13]. A probability (*p*) value ≤ 0.05 was used to determine the significance of difference.

### 3.8. The Effect of NADPH Oxidase Inhibitor on Sclerotial Formation by Temperature-Shift Treatment

Before inoculation, DPI was added to the medium in a final concentration of 1 mmol L^−1^, 2 mmol L^−1^, 3 mmol L^−1^. Sclerotial formation was observed during the cultivation period after the antioxidant was added. At the same time, the antioxidant enzymes SOD and CAT were detected respectively at each cultivation stage.

### 3.9. Mycelial and Sclerotial Protein Extraction and Concentration Determination

The fresh sclerotia and mycelia were separated by using sterile forceps and washed thrice in two times volumes of ice-cold phosphate-EDTA buffer [24]. They were collected at different cultivating phases and put into the mortar. Each sample was mixed with a good amount of liquid nitrogen and ground to a powder quickly. Then, 0.8 mL of PBS (phosphate buffer solution) was added to each sample (0.05 mol L^−1^, pH 7.2) and transferred to the centrifuge tube, centrifuged at 20, 000× *g* for 20 min, and the supernatant (cytoplasmic fraction) was collected and used for the following assays. This supernatant was considered to be the crude enzymic solution.

The reaction mixture with a total volume of 405 μL containing 2.5 μL of crude enzyme of the mycelia or the sclerotia, 202.5 μL of PBS and 200 μL of BCA was incubated at 20 °C for 60 min, and the absorbance at 562 nm was measured by using the Bio-Rad Smart spec™ plus spectrophotometer (Bio-Rad Corporation, Hercules, CA, USA). The protein content was calculated according to the standard curve. The experiment was repeated three times.

The protein concentration was detected by protein quantitative reagent kit using the BCA (bovine serum albumin) method [25]. The BCA protein assay kit was the product of Merck of Novagen (New York, NY, USA). The BCA reactive solution with pH 11.25 contains 0.1 M of bicinchoninic acid, NaOH buffer, sodium carbonate, sodium tartrate and sodium bicarbonate. Firstly, a BCA working solution composed of BCA reactive solution and 4% copper sulfate was prepared as follows: BCA reactive solution was shaken evenly. The volume of BCA reactive solution was 50 times that of 4% copper sulfate and they were mixed together evenly. Secondly, after the bovine serum albumin (BSA) of 2 mg mL^−1^ as the protein standard solution was dissolved, 250 μL of BSA (2 mg mL^−1^) was diluted twice by using 250 μL of BCA reactive solution, to give the final concentration of BSA as 1 mg mL^−1^. The BSA of 1 mg mL^−1^ was diluted by the method mentioned above. The dilution work should be repeated until BSA of 25 μg mL^−1^ was prepared. Fifty microliters of each group of the diluted BSA of 1000 μg mL^−1^, 500 μg mL^−1^, 250 μg mL^−1^, 125 μg mL^−1^, 25 μg mL^−1^ and 0 μg mL^−1^ were added to the colorimetric cup respectively. One mL of the BCA working solution was also added to the same cups with BSA. Finally, the mixture solution was added to each colorimetric cup and incubated at 37 °C for an hour. Then the absorbance at 562 nm was measured using the Bio-Rad Smart spec™ plus spectrophotometer, and the standard curve was drawn. The samples were measured in the same way as for the detection of the BSA standard curve.

### 3.10. SOD Detection during *P. umbellatus* Sclerotial Formation

The method is based on the SOD-inhibited reduction of oxidized dianisidine according to previous studies [26,27], which results from reaction of its reduced form by photochemically sensitized riboflavin. The reduced riboflavin can be easily oxidized to generate O_2_^−^ with oxygen. O_2_^−^ can reduce nitroblue tetrazolium (NBT) to blue methyl hydrazine with absorbance maximum at 560 nm. This process is antagonized by SOD. The assay consists of the following reagents: 1.5 mL of 0.05 mol/L phosphate buffer, 0.3 mL of 130 mmol/L methionine, 0.3 mL of 750 μmol/L NBT, 0.3 mL of 100 μmol/L EDTA–Na_2_, 0.3 mL of 20 μmol/L riboflavin and 0.05 mL of crude enzyme solution. In the control group, the phosphate buffer was replaced with crude enzyme. One of the tubes in the control group as blank was placed in the dark, and the other tubes were put in the light with 4000 lux for 20 min. After the reaction finished, the absorbance was detected and recorded in all reactive tubes. One SOD unit was defined as 50% of the suppression of the NBT photochemical reduction. The absorbance measurements were done in a Bio-Rad Smart spec™ plus spectrophotometer. The experiment was repeated three times, with 10 replicates in each group.

### 3.11. CAT-Dependent H_2_O_2_ Consumption Assay during *P. umbellatus* Sclerotial Formation

The assay was based on the CAT detection kit made by Biological Engineering Institute, Nanjing, China. Briefly, CAT decomposes H_2_O_2_ but can be rapidly interrupted by molybdic acid. The residual H_2_O_2_ and molybdic acid can form a light yellow complex which has the largest absorbance at 405 nm, thus the CAT activity can then be calculated. The absorbance measurements were taken in a Bio-Rad Smart spec™ plus spectrophotometer. One specific activity of catalase is defined as each micromole H_2_O_2_ consumed by enzyme in one minute. One unit of CAT is how much H_2_O_2_ is decomposed by each gram of fresh sclerotia or mycelia in every minute. *P. umbellatus* cultivated at 25 °C was designated as the control group. The fungus cultivated at 8 °C was named as the experiment group. The experiment was repeated three times, with 30 replicates in each group.

### 3.12. Data Analysis

The data were analyzed with one-way ANOVA; all statistical analyses were performed using SPSS 11.0 (SPSS, Chicago, IL, USA). Significant differences were determined using the Student–Newman–Keuls method. *p* values <0.05 were considered significant.

## 4. Conclusions

In this study, cytochemical localization of ROS generation at the subcellular level during *P. umbellatus* sclerotial formation was detected. Furthermore, the *Nox* gene expression in the sclerotia and mycelia was analyzed for the first time by using qRT-PCR. Our results elucidated the important roles of *Nox* in *P. umbellatus* sclerotial formation induced by temperature shift. The specific activity of SOD and CAT detection indicated that antioxidant enzymes were involved in cellular stress responses and anti-oxidative processes. In order to gain a better understanding of the molecular mechanism of *P. umbellatus* sclerotial formation, more future studies will be necessary to investigate the functions of the *Nox* gene and the more differentially expressed genes of the sclerotia and the mycelia.
